# Black box modeling of PIDs implemented in PLCs without structural information: a support vector regression approach

**DOI:** 10.1007/s00521-014-1754-2

**Published:** 2014-10-26

**Authors:** Robert Salat, Michal Awtoniuk

**Affiliations:** Department of Production Engineering, Warsaw University of Life Sciences, Nowoursynowska 166, 02-787 Warsaw, Poland

**Keywords:** Support vector regression, Programmable logic controller, PID

## Abstract

In this report, the parameters identification of a proportional–integral–derivative (PID) algorithm implemented in a programmable logic controller (PLC) using support vector regression (SVR) is presented. This report focuses on a black box model of the PID with additional functions and modifications provided by the manufacturers and without information on the exact structure. The process of feature selection and its impact on the training and testing abilities are emphasized. The method was tested on a real PLC (Siemens and General Electric) with the implemented PID. The results show that the SVR maps the function of the PID algorithms and the modifications introduced by the manufacturer of the PLC with high accuracy. With this approach, the simulation results can be directly used to tune the PID algorithms in the PLC. The method is sufficiently universal in that it can be applied to any PI or PID algorithm implemented in the PLC with additional functions and modifications that were previously considered to be trade secrets. This method can also be an alternative for engineers who need to tune the PID and do not have any such information on the structure and cannot use the default settings for the known structures.

## Introduction

Proportional–integral–derivative (PID) algorithms are widely used for the control of industrial process loops. Due to their simplicity and ease of on-line re-tuning, approximately 90 % of control loops use the PID algorithm. Among the control loops that use the PID algorithm, 64 % are single loop and 36 % are multi-loop [1]. Approximately 85 % of control systems that use PID algorithms are the feedback type, up to 6 % are the feedforward type, and 9 % are connected in a cascade [1, 2]. PID algorithms implemented as a technical device are called PID controllers. Currently, the PID controller structures are different from the original analogue PID [3]. Presently, the implementation of the PID is based on a digital design. These digital PID controllers include several additional functions to improve their performance, such as anti-windup, set point filtering, auto-tuning, adaptive algorithms, fuzzy fine-tuning, genetic tuning, and so on [4]. The controllers come in several different forms, such as a standard single-loop controller, known as a dedicated process controller and a software component in the programmable logic controller, known as a programmable logic controller (PLC) [5], as well as in built-in controller machines, e.g., robots [6]. PID controllers are used in a wide range of application, such as process control, flight control, automotive control, motor drives, and so on. The PID algorithms found in industry may have different structures [3]. Currently, the three largest classes of PID algorithms implemented in the PLC are the ideal standard algorithm (ISA, non-interacting), parallel (non-interacting), and series (interacting) types. The general expressions of the PID algorithms are represented by (1, 2, 3) as follows:

1 $$u = K_{\text{C}} \left( {1 + \frac{1}{{T_{\text{I}} s}} + T_{\text{D}} s} \right)$$

2 $$u = K_{\text{P}} + \frac{{K_{\text{I}} }}{\text{s}} + K_{\text{D}} s$$

3 $$u = K_{\text{C}}^{{\prime }} \left( {\frac{1}{{T_{\text{I}}^{{\prime }} s}} + 1} \right)\left( {T_{\text{D}}^{{\prime }} s + 1} \right)$$

where *K*_C_ is the proportional gain of the ISA; *T*_I_ is the reset time of the ISA; *T*_D_ is the derivative time of the ISA; *K*_P_ is the proportional gain of the parallel form; *K*_I_ is the integral gain of the parallel form; *K*_D_ is the derivative gain of the parallel form; $K_{\text{C}}^{{\prime }}$ is the proportional gain of the series form; $T_{\text{I}}^{{\prime }}$ is the reset time of the series form; and $T_{\text{D}}^{\prime }$ is the derivative time of the series form.

Previously, the parallel and ISA algorithms were less commonly observed in industrial processes compared to the series form. The reason for this phenomenon can be found in analog control, where pneumatic controllers were dominant. When pneumatic controllers were dominant, the PID algorithm was difficult to design due to its use of extremely expensive analog amplifiers [7]. Despite this drawback, Astrom and Hagglund [2] indicated that the ISA allows complex zeroes and is thus a more flexible structure than the series algorithm, which has real zeroes. Many controller manufacturers (ABB, Allen-Bradley, General Electric, Honeywell, Omron, Siemens, Toshiba, Yokogawa, etc.) offer a variety of modified versions of the above-mentioned forms, where a few of the modifications are improvements of the structures, a few of the modifications are derived from early pneumatic implementations, and a few of the modifications are more common in certain industries than others. According to [2], many useful features of PID control have not been widely disseminated because they are considered to be trade secrets. Typical examples include techniques for mode switches and anti-windup. However, the basic actions remain the same. The main issue is that the tuning behavior varies from one form to another. An understanding of the various forms of PID algorithms and the configuration options that are offered is necessary to properly design and apply process control strategies. For this type of modified structure, there are no readily available tuning rules [8, 9]. Rhinehart and Shinskey [3] reported that an operator who was accustomed to tuning a controller with a particular PID algorithm would be baffled when another controller did not respond as expected. An operator or software program that follows a tuning procedure to determine *K*_C_, *K*_P,_ or $K_{\text{C}}^{{\prime }}$; *T*_I_, *K*_I,_ or $T_{\text{I}}^{{\prime }}$; and *T*_D_, *K*_D,_ or $T_{\text{D}}^{\prime }$ for the standard algorithm (1, 2, 3) could be surprised by the response when applying the procedure to a manufacturer-specific version.

Therefore, a PID controller’s structure should be completely understood before it is tuned [10]. Furthermore, the structural difference becomes significant when one controller is replaced by another. A variety of structure identification methods are under development. A popular and frequently used method is the relay feedback for both the off-line and on-line automatic identification of the PID [7]; however, relay feedback has disadvantages because it is unacceptable for a few classes of processes, such as unstable and integrals of the second-order processes. A different approach to identify PI structures was shown in [11], where artificial intelligence was used and PI algorithms were treated as a black box [12].

In this report, we propose using the support vector regression (SVR) [e.g., the support vector machine (SVM) in regression mode] as a tool for PID-implemented modeling in a real PLC. According to [8], about fifty non-standard PID structures very often can be seen in the real PLC. Thus, this method can be an alternative for engineers who need to tune the PID and do not have any such information on the structure and cannot use the default settings for the known structures. Preliminary research for this method was presented in [11] and was limited to the PI algorithm.

In the first stage, we used the SVR for training PID algorithm structures based on input and output signals from the PLC (black box model). The goal was to train a SVR and obtain the response comparable to the response of the PID algorithm implemented in the PLC for any parameters *K*_P_, *T*_I_, and *T*_D_. The advantage of this method is that the structures with modification can then be both known and unknown. The benefits of this method also indicate that after the training of the SVR, we can simulate the real output of the PID algorithm for a personal computer (PC) and apply, for example, an imperialist competitive algorithm (ICA) to tune the PID parameters and transfer them to the PLC.

We used the SVR because it is a good tool to estimate regression functions with generalization performances when using structural risk minimization [13]. One of the problems of using the SVR is that a large number of samples are gathered with the PLC in addition to a few of the features selected in mode training and testing. In this report, we propose the selection of an optimal feature vector and reduction samples to be trained. We focus on two industrial controllers: Siemens and General Electric (GE), the basic algorithms of which have already been described (1, 2). To the best of our knowledge, SVR has never been used in the context of PID algorithms implemented in the PLC.

The method is sufficiently universal and can be applied to any PI, PD, and PID algorithm implemented in the PLC, with additional functions and modifications that are considered to be trade secrets. The report is organized as follows. The structures of the PID algorithms implemented in the PLC are described in Sect. 2. The short studies on the SVR are described in Sect. 3. The proposed feature selection for the SVR is described in Sect. 4. Section 5 is devoted to describing the results. Finally, conclusions and future studies are provided.

## Structures of PID algorithms that are most commonly implemented in PLC

Many controller manufacturers offer a variety of PID versions. It is important to remember that there is no standard terminology used among manufacturers. To reveal the controller’s structure, the user should examine the mathematical expression included in the user manual rather than relying on the manufacturer’s nomenclature.

PID algorithms that continuously or repetitively calculate the required position of the valve or other final actuator are called position algorithms. Conversely, algorithms that calculate the required change in position of the final actuator are called velocity or incremental algorithms. The position algorithm is the most popular algorithm in PLCs. Three basic PID structures are described below.

### PID structures

**Fig. 1:**
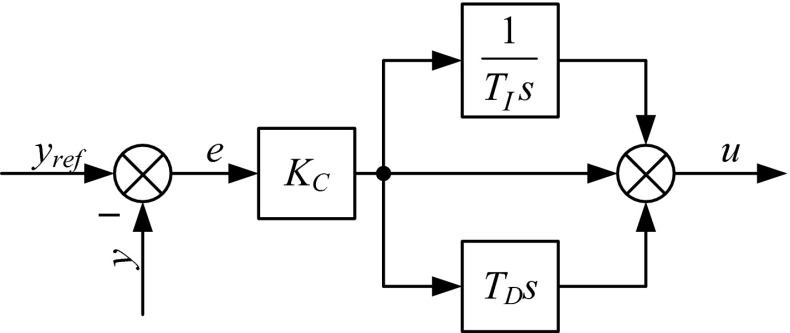
Block diagram of an ISA PID controller

The first controller structure, which is most often implemented, is called the ISA. This form is also labeled as the dependent or gain-dependent form. The controller output is calculated as presented in (2). The block diagram of the ISA PID is shown in Fig. 1, where *y*_ref_ is set point, *y* is measurement, *e* is control error, *u* is control variable.

The ISA is used in numerous controllers, e.g., GE VersaMax PLC (‘PID ISA’ function block), Siemens S7-300 (‘FB41’ function block), and Allen-Bradley PLC-5 (‘PID’ function block) [14, 15].

In this PID, *K*_C_ is dimensionless, the units of *T*_I_ are minutes per repeat, and the units of *T*_D_ are minutes. However, various manufacturers express *T*_I_ and *T*_D_ in seconds rather than minutes.

In certain cases, the proportional action can be expressed as a proportional band (PB) rather than a proportional gain, *K*_C_. The integral mode can be described by the reset time, *T*_I_, or reset rate, *T*_R_. The units of *T*_R_ are repeats/minute.

**Fig. 2:**
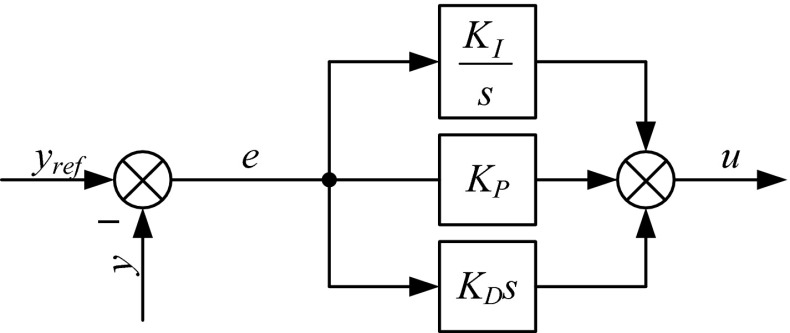
Block diagram of a parallel PID controller

The next PID structure, described by (2), is parallel, ideal parallel, independent, or gain independent. The proportional gain, *K*_C_, is dimensionless; the integral gain, *K*_I_, is expressed in units of time^−1^; and the derivative gain, *K*_D_, is expressed in units of time. Examples of this form are the ‘PID IND’ function block in the GE VersaMax PLC and ‘PID’ function block in the Allen-Bradley PLC-5 [16]. Figure 2 illustrates how the controller output is calculated.

**Fig. 3:**
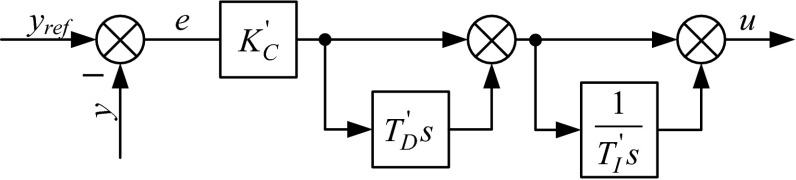
Block diagram of a series PID controller

Equation (3) and Fig. 3 refer to the series controller structure. This form should be considered as interacting because the integral block is in series with the derivative block, and if one of the blocks change, the other block is affected. Using this nomenclature, the parallel and ISA form should be classified as non-interacting structures because the integral and derivative blocks have a parallel connection. It is worth noting that if the integral or derivative term is turned off, then the series and ISA forms are identical. The units of the controller’s parameters, $K_{\text{C}}^{{\prime }}$, $T_{\text{I}}^{{\prime }}$, and $T_{\text{D}}^{\prime }$, are the same as the ISA’s parameters. An example of this form is the Foxbro controller [8].

### Additional function and modification

**Fig. 4:**
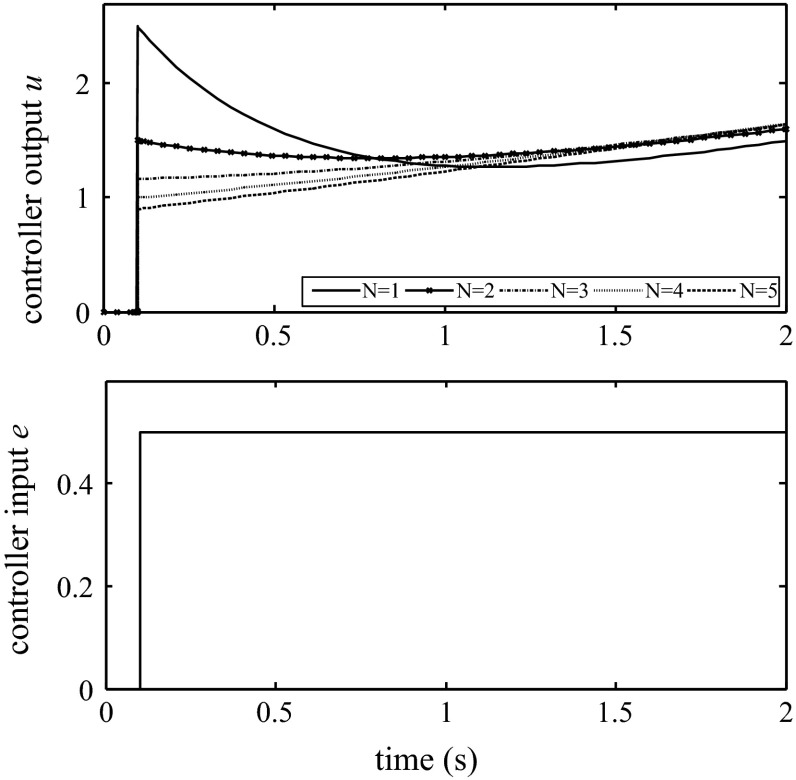
Influence of the *N* value on controller dynamics; *K*_C_ = 1; *T*_I_ = 1; *T*_D_ = 0.5; ISA form

In certain cases, a sudden set point change may cause a spiking reaction in the controller output. To prevent this phenomenon, numerous manufacturers propose an additional parameter that can weaken the proportional action and soften the response of the set point. Another approach to allow the controller output to be gentler is to add a derivative smoothing filter. Equation (4) describes the ISA controller with an embedded derivative filter. The coefficient *N* has a significant impact on the controller dynamics. There is no standard value of this coefficient among manufacturers, e.g., in the Siemens S7-300 and Toshiba T-series, the value of *N* is equal to 10, whereas in the Allen-Bradley PLC-5, the value of *N* is equal to 16 [16, 17]. Figure 4 shows the manner in which the *N* value can influence the controller dynamics. In the GE VersaMax PLC, the user can also enable derivative filtering by applying a first-order filter [15]. Unfortunately, manufacturers do not describe this filter in great detail.

4 $$u = K_{\text{C}} \left( {1 + \frac{1}{{T_{I} s}} + \frac{{T_{\text{D}} s}}{{1 + \frac{{T_{\text{D}} }}{N}s}}} \right)$$

**Fig. 5:**
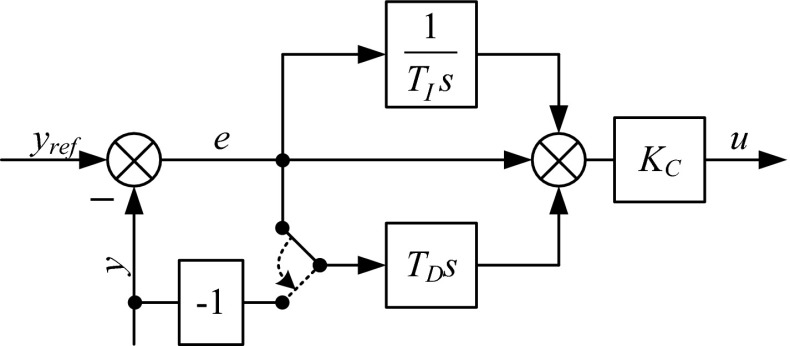
ISA form with a switchable derivative action

**Fig. 6:**
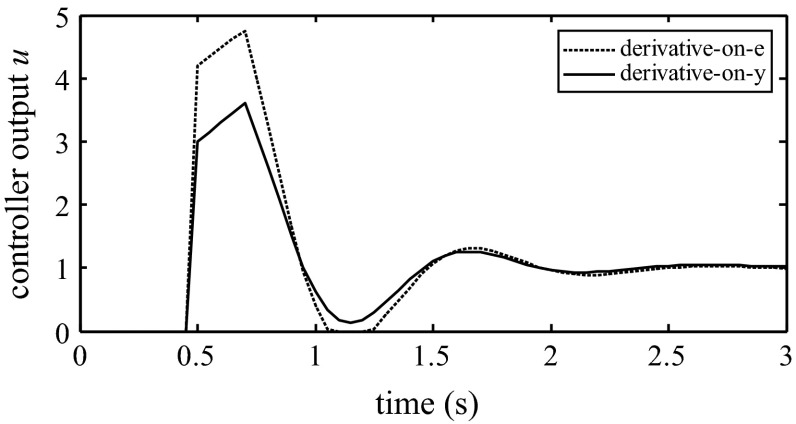
Comparison of derivative on measurement and derivative on error

Furthermore, the PID structure can be modified, so that the derivative term acts only on the y signal. In most PLCs, this modification is a user option. This modification can be performed by switching the proper bit or register in the PLC’s memory. This result contributes to the elimination of controller output bumps. The implementation of this option is shown in Fig. 5. Figure 6 shows a comparison of the derivative-on-measurement and derivative-on-error PID structures.

A popular addition is the deadband. This is the quantity that is compared to the error signal. If the error is within the deadband range, an update of the controller output does not occur.

Another problem strongly connected with PI and PID controllers is an integral or reset windup. This problematic situation occurs when the controller output signal remains at its maximum or minimum limit, even though the value of the error begins to decrease or increase. The integrator windup can be avoided by verifying that the integral is kept at a proper value when the controller’s output saturates; thus, the controller is ready to resume action as soon as the error changes. Furthermore, there are several solutions for reset windup problems, but in practice, manufacturers of PLCs do not describe which solution they use.

## SVR studies

Support vector machines (SVMs) are classification and regression methods, and the basis of these methods has been derived by Vapnik and Chervonenkis [18]. SVMs that address classification problems are called support vector classifications (SVCs) [19], and SVMs that address modeling and prediction are called SVRs [13]. The purpose of the SVR is to obtain a function with a maximum deviation of *ε* from the actual destination vectors for all given training data that is as flat as possible. SVR requires the setting of fewer user-defined parameters as well as the option of a kernel and its parameters. The advantage of SVR over conventional algorithms based on empirical risk minimization, such as an artificial neural network, is its optimization algorithm, which includes solving a linearly constrained quadratic programming function, leading to an optimal and global solution. SVR has been successfully used to solve problems in many fields, such as economics [20], medicine [21], electrical circuits [22], power systems [23], mechanics [24], and system identification [25, 26].

Let us assume that we have a data set of *p* training samples, $\left\{ {\left( {x_{1} ,d_{1} } \right),\left( {x_{2} ,d_{2} } \right), \ldots ,\left( {x_{p} ,d_{p} } \right)} \right\}$, where ${\mathbf{x}}_{i} \in R^{n} , \, d_{i} \in R$. We can introduce a nonlinear mapping $\varphi \left( \cdot \right) :\;R^{n} \to H$, where *H* is a hypothetical feature space, and define *ε*—insensitive loss function—as follows:

5 $$L_{\varepsilon } = \left| {d - y({\mathbf{x}})} \right|_{\varepsilon } = { \hbox{max} }\left\{ {0,\left| {d - y({\mathbf{x}})} \right| - \varepsilon } \right\}$$

where *y*(**x**) is the estimation of the function. The SVR formula can be expressed as follows:

6 $$y({\mathbf{x}}) = {\mathbf{w}}^{T} \varphi (x) + b\quad {\mathbf{w}},{\mathbf{x}} \in R^{n} \quad b \in R$$

where **w** is the weight vector and *b* is the offset.Then, *y*(**x**) can be determined from the minimization problem as follows:

7 $$\hbox{min} \; \, L_{\varepsilon } = \hbox{min} \frac{1}{p}\sum\limits_{i = 1}^{p} {\left( {\left| {d_{i} - {\mathbf{w}} \cdot \varphi ({\mathbf{x}}_{i} ) - b} \right| - \varepsilon } \right)}$$

By introducing slack variables *ξ*_*i*_, *ξ*_*i*_ * into (7), an optimization problem can be formulated as follows:

8 $$\mathop {\hbox{min} }\limits_{{w,b,\xi ,\xi_{i}^{*} }} \, \frac{1}{2}\left\| {\mathbf{w}} \right\|^{2} + C\mathop \sum \limits_{i = 1}^{p} \xi_{i} + C\mathop \sum \limits_{i = 1}^{p} \xi_{i}^{*}$$

which is subject to:

9 $$\begin{aligned} & d_{i} - {\mathbf{w}}^{T} \varphi \left( {{\mathbf{x}}_{{\mathbf{i}}} } \right) - b \le \varepsilon + \xi_{i} \\ & {\mathbf{w}}^{T} \varphi \left( {{\mathbf{x}}_{{\mathbf{i}}} } \right) + b - d_{i} \le \varepsilon + \xi_{i}^{*}\quad \xi_{i} ,\xi_{i}^{*} \ge 0 \end{aligned}$$

The constant C > 0 determines the trade-off between the model flatness and the training error. The flatness in (6) indicates a small **w** value.

The solution to the optimization problem in (8) is given by the saddle point of the Lagrangian as follows:

10 $$\begin{aligned} J\left( {w,\xi ,\xi ,^{*} \alpha ,\alpha^{*} ,\gamma ,\gamma^{*} } \right) & = \frac{1}{2}\left\| {\mathbf{w}} \right\|^{2} + C\mathop \sum \limits_{i = 1}^{p} \xi_{i} + C\mathop \sum \limits_{i = 1}^{p} \xi_{i}^{*} \\ & \quad - \mathop \sum \limits_{i = 1}^{p} \alpha_{i}^{*} \left( {d_{i} - {\mathbf{w}}^{T} \varphi \left( {{\mathbf{x}}_{{\mathbf{i}}} } \right) - b + \varepsilon + \xi_{i}^{*} } \right) \\ & \quad - \mathop \sum \limits_{i = 1}^{p} \alpha_{i} \left( {{\mathbf{w}}^{T} \varphi \left( {{\mathbf{x}}_{{\mathbf{i}}} } \right) + b - d_{i} + \varepsilon + \xi_{i} } \right) - \mathop \sum \limits_{i = 1}^{p} \left( {\gamma_{i} \xi_{i} + \gamma_{i}^{*} \xi_{i}^{*} } \right) \\ \end{aligned}$$

It follows from the saddle point condition that the partial derivatives of *J* with respect to the primal variables (***w***, *ξ*_*i*_, *ξ*_*i*_ *) must be excluded for optimality. The variables *α*_*i*_, *α*_*i*_ *, *γ*_*i*_, *γ*_*i*_ * must satisfy the positivity constraints. The formulation of the dual problem involving the Lagrange multiplier *α* is equivalent to finding an expression as follows:

11 $$\mathop {\hbox{min} }\limits_{{\alpha ,\alpha^{*} }} \, \frac{1}{2}\left( {\alpha - \alpha^{*} } \right)^{T} Q\left( {\alpha - \alpha^{*} } \right) + \varepsilon \mathop \sum \limits_{i = 1}^{p} \left( {\alpha_{i} + \alpha_{i}^{*} } \right) + \mathop \sum \limits_{i = 1}^{p} d_{i} \left( {\alpha_{i} - \alpha_{i}^{*} } \right)$$

which is subject to:

12 $$\mathop \sum \limits_{i = 1}^{p} \left( {\alpha_{i} - \alpha_{i}^{*} } \right) = 0\quad 0 \le \alpha_{i} ,\alpha_{i}^{*} \le C$$

where $Q_{ij} = k({\mathbf{x}}_{i} ,{\mathbf{x}}_{j} ) = \varphi^{T} ({\mathbf{x}}_{i} )\varphi ({\mathbf{x}}_{j} )$ is the kernel function in accordance with Mercer’s condition [27]. The kernel function has been defined as a linear dot product of the nonlinear mapping.

After solving the problem in (8), the regression function can be written as follows:

13 $$y\left( {\mathbf{x}} \right) = \mathop \sum \limits_{j = 1}^{K} (\alpha_{i}^{*} - \alpha_{i} )k({\mathbf{x}},{\mathbf{x}}_{i} ) + b$$

where *K* is the number of so-called support vectors (SV). The vector **x**_*i*_ is associated with the coefficient *α*_*i*_ is called a support vector, and only those vectors have an effect on *y*(**x**).

The selection of the coefficients *ε* and C is of utmost importance. The constant *ε* determines the margin within which the error is neglected. The smaller its value, the more support vectors will be determined by the algorithm. The constant C is the weight, which determines the trade-off between the complexity of the network, characterized by the weight vector and the error of approximation and is measured by the slack variables (*i* = 1, 2, …, *p*).

## Data collection

The preparation of training data and feature selection (i.e., a model selection) is usually the most important factors influencing the correct operation of the model and the ability to generalize the SVR. To generate a training and validation data set for the identification of the PID algorithm, an experiment is performed. In our study, a validation term is different than a test term and is explained in the next section.

**Fig. 7:**
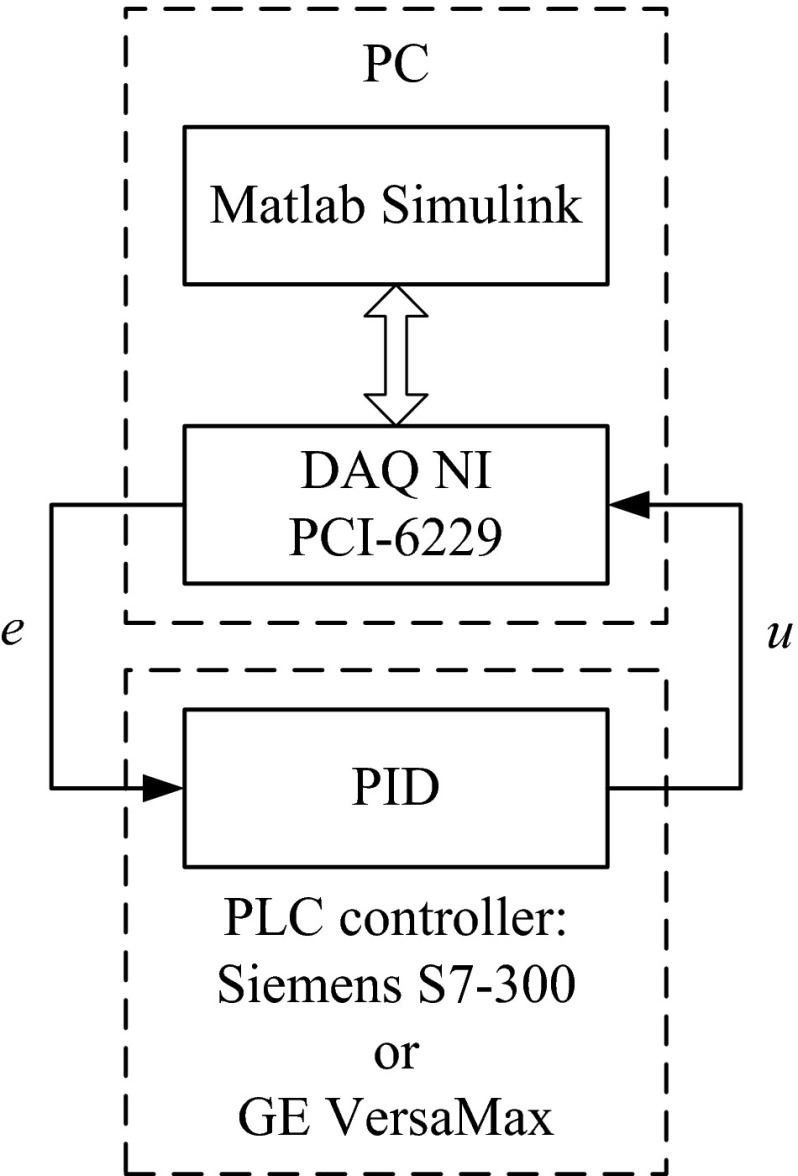
Block diagram of data set collection

The training and validation data sets collected by the system for our study are shown in Fig. 7.

However, there are difficulties with training the SVR on a real data set. As the number of training patterns increases, the generation of the SVR training takes significantly longer, with a time complexity of *p* ^3^, where *p* is the number of training patterns. Thus far, several algorithms, such as chunking, SMO, SVM light, and SOR, have been proposed to reduce the training time [28].

The control error, *e*, and control variable, *u*, of the PID algorithm have been administered and recorded using a PC with Matlab and Simulink 7.10.0 (R2010a) MathWorks. In the Matlab and Simulink toolbox, the real-time windows target was used. The connection between the PLC and PC was maintained through a National Instruments data acquisition board, NI PCI-6229. The sampling frequency of both the PLC and Matlab & Simulink was 10 Hz (recommended frequency by the manufacturer of the PLC).

Many attempts have shown that better SVR training results were obtained by artificial excitation signals of input rather than trying to collect data from a process that is under a closed-loop control. When the input–output patterns come from a PID under loop control, the variables are highly correlated with each other, and the information content is low. Most sampled states would reside in a narrow region around the operating point, giving minimal information on the interaction of the different input variables to produce the output [29]. Therefore, the control error, *e*, was administered in the form of uniformly distributed random signals of a different amplitude and frequency in the interval of −1 to 1 V. This type of artificial excitation should be selected so as to not turn on anti-windup because it will be tested at a later stage.

**Fig. 8:**
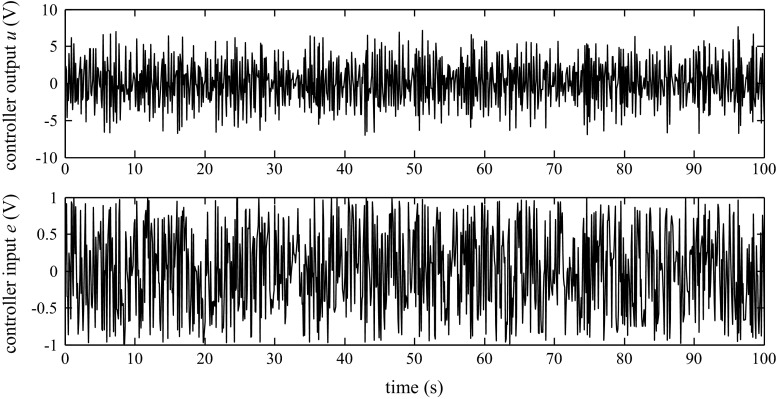
Example of a signal input and output of a PID algorithm for *K*_P_ = 0.47, *K*_I_ = 5.88, *K*_D_ = 0.08 (PLC GE VersaMax ‘PID IND’ function block)

The example of a signal *e* and *u* collected for training and validation of the SVR is shown in Fig. 8.

As a result of performing simulations for different settings of the PID algorithm (PLC Siemens, GE) with different values of amplitude and frequency of the control error, *e*, administered on the input of the PID, hundreds of thousands of training samples were collected.

## Model selection

Because the SVR has to emulate the work of the PID algorithm, the goal of this study is to construct a multiple-input and single-output (MISO) black box model for the output of the PID algorithms. The input structure of the SVR was used in accordance with the NARX (Nonlinear AutoRegressive with eXogenous inputs) model in a predictor form. To obtain a good NARX model, the selection of the amount of regressors is extremely important [30, 31]. The regressors are the inputs of the model. In our case, the output of the model depends on past inputs and outputs and can be described as follows:

14 $$\begin{aligned} \hat{u}(t) & = f({\mathbf{\varphi }}(t),\varvec{\theta}), \\ {\mathbf{\varphi }}(t) & = [e(t),e(t - 1), \ldots e(t - n_{e} ),u(t - 1), \ldots u(t - n_{u} ))] \\ \end{aligned}$$

where $\hat{u}(t)$ is the output of the model; ***φ***(*t*) is the regression vector; ***θ*** is the parameter vector; and *n*_*e*_ and *n*_*u*_ indicate the order of the NARX model (number of lags).

Finally, the set of SVR inputs (features input set) is extended with *P*, *I*, and *D* parameters, and the general form is as follows:

15 $${\mathbf{x}} = \left[ {P\;I\;D\;e(t) \, e(t - 1) \ldots e(t - n_{e} ) \, u(t - 1) \ldots u(t - n_{u} )} \right]$$

where *P* = *K*_C_ for the ISA; *P* = *K*_P_ for the parallel form; *I* = *T*_I_ for the ISA; *I* = *K*_I_ for the parallel form; *D* = *T*_D_ for the ISA; *D* = *K*_D_ for the parallel form; *e* is the control error at time *t*, *t* − 1, …, *t* − *n*_*e*_; and *u* is the controller output at time *t* − 1, …, *t* − *n*_*u*_.

The output signal of the SVR was the control variable $\hat{u}(t)$.

**Fig. 9:**
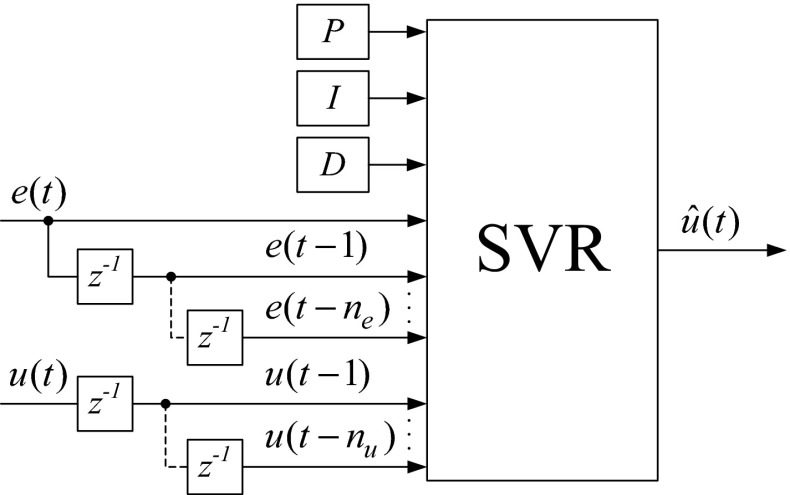
General block diagram of the input vector and the output of the SVR

The general block diagram of the full set feature vector covering both input and output is shown in Fig. 9.

## Results

The SVR was trained and tested for the PID algorithms, which were implemented in S7-300 Siemens and GE Versa Max controllers. The PID algorithms implemented in the controllers’ PLC were ISA (Siemens, GE) and parallel (GE). The manufacturers provided the transfer function over a discrete time, described in (16) for the parallel form and (17) for the ISA, as follows:

16 $$u = K_{\text{P}} + K_{\text{I}} T_{\text{S}} \frac{1}{z - 1} + K_{\text{D}} \frac{N}{{1 + NT_{\text{S}} \frac{1}{z - 1}}}$$

17 $$u = K_{\text{C}} \left( {1 + \frac{{T_{\text{S}} }}{{T_{\text{I}} }}\frac{1}{z - 1} + T_{\text{D}} \frac{N}{{1 + NT_{\text{S}} \frac{1}{z - 1}}}} \right)$$

where *T*_S_ is the sampling time.

There is no information on the value of *N* or the modification of the structure. After many attempts, a value of *N* equal to 10 was chosen for all structures.

The results of the SVR with the neural network NARX model (NN) and transfer function (*T*_f_) of the PID described by the manufacturers (16, 17) were compared. For NN structure, selection was made according to the procedure proposed for the SVR. Number of both inputs and outputs was the same as for the SVR. The initial number of neurons in the hidden layer was assumed as the root of the sum of inputs and outputs and then fine-tuned by trial and error method. The learning for NN was performed using Levenberg–Marquardt Method. The NN and Tf were implemented in Matlab & Simulink 7.10.0 (R2010a). More detailed description of the mechanism of the NN can be found in [32].

The data set was divided into the training set and validation set without averaging or filtering. The first 5 % of the data was used for estimation, and the last 95 % was used for validation. In the training and validation mode, a one-step-ahead prediction was performed, whereas in the test mode, the simulation (the measured inputs and estimated outputs are used to form the regressors) was performed. The differences between the validation and test mode are due to the lack of implementing the cross-validation function in the SVR toolbox.

**Fig. 10:**
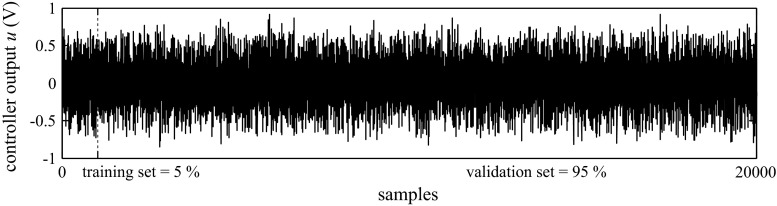
Set of samples for different settings for both the training and validation mode

The data set for training and validation for each of these structures was generated for dozens of different settings. The data set was normalized over the range of 0–1. The ranges for each setting were as follows: *K*_C_: 0.5–0.9; *T*_I_: 0.5–9.9; *T*_D_: 0.03–0.48; *K*_P_: 0.1–0.47; *K*_I_: 0.1–0.91; and *K*_D_: 0.01–0.5. A sample set for different settings is shown in Fig. 10.

Quality measures for the training, validation, and testing set are performed on the basis of the mean squared error (MSE) and fit measure (Fit) methods.

The mean squared error (MSE) is defined as follows:

18 $${\text{MSE}} = \frac{{\left\| {u(t) - \hat{u}(t)} \right\|_{2}^{2} }}{p}$$

The Fit measure is defined as follows:

19 $${\text{Fit}} = \left( {1 - \frac{{\left\| {u(t) - \hat{u}(t)} \right\|_{2} }}{{\left\| {u(t) - \bar{u}(t)} \right\|_{2} }}} \right) \times 100\;\%$$

where $\hat{u}(t)$ is the simulated output; *u*(*t*) is the measured output; and $\bar{u}(t)$ is the mean of the measured output; *p* is the number of samples.

**Fig. 11:**
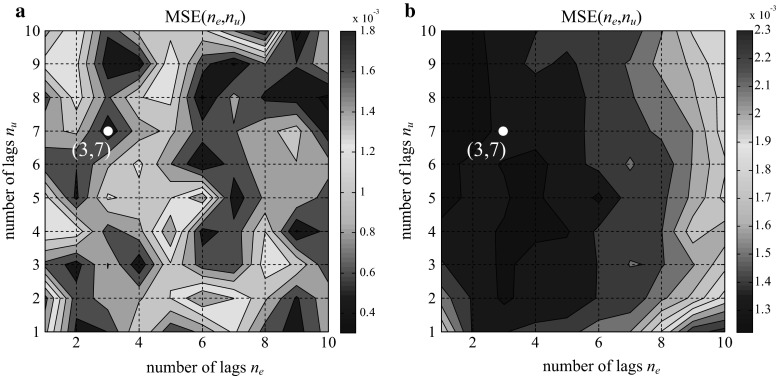
MSE of the training data (**a**). MSE of the validation data (**b**); parallel form GE

**Fig. 12:**
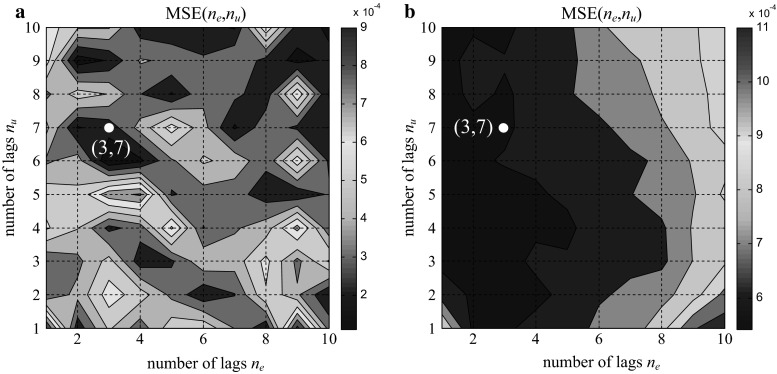
MSE of the training data (**a**). MSE of the validation data (**b**); ISA form GE

**Fig. 13:**
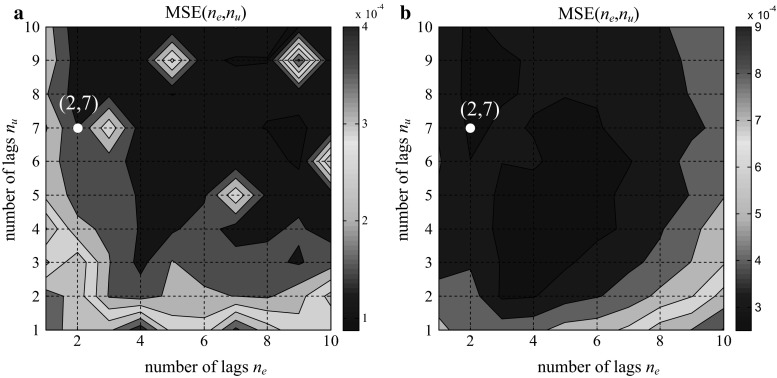
MSE of training data (**a**). MSE of the validation data (**b**); ISA form Siemens

To define the optimal number of regressors in the training data set and the impact of the number of regressors on the model, a training approach of trial and error was performed. Because the order of the transfer function of the PID algorithm was low, the number of lags was determined over the range of 1–10. The minimum MSE values of the analysis of all possible combinations in relation to *n*_*u*_: 1–10 and *n*_*y*_: 1–10 for the three structures are plotted in Figs. 11,12, and 13.

The observation of the MSE values shows that there is no clear minimum in the above figures, which indicates the possibility that the model class is not entirely correct. Attempts to determine the minimum value or a point close to the minimum value for both the training data and validation were performed. We also tried to find a compromise between the MSE index and the size of the input vector. The effect of the selected values on the results of the test data was also observed.

Finally, based on the MSE method for the training and validation data set, optimal values of (3, 7), (3, 7), and (2, 7) were chosen, as depicted in Figs. 11, 12, and 13, respectively.

For those values, the feature vectors of the SVR were prepared as follows:

Parallel structure GE:

20 $${\mathbf{x}} = \left[ {P\;I\;D\;e(t) \, e(t - 1) \ldots e(t - 3) \, u(t - 1) \ldots u(t - 7)} \right]$$

ISA structure GE:

21 $${\mathbf{x}} = \left[ {P\;I\;D\;e(t) \, e(t - 1) \ldots e(t - 3) \, u(t - 1) \ldots u(t - 7)} \right]$$

ISA structure Siemens:

22 $${\mathbf{x}} = \left[ {P\;I\;D\;e(t) \, e(t - 1) \, e(t - 2) \, u(t - 1) \ldots u(t - 7)} \right]$$

After the feature vectors were selected, the training process of the SVR was performed using an SMO-type algorithm implemented in the toolbox LIBSVM [33]. We used the Gaussian radial basis function as the kernel function $k({\mathbf{x}},{\mathbf{x}}_{\text{i}} ) = e^{{ - \gamma \left\| {{\mathbf{x}} - {\mathbf{x}}_{\text{i}} } \right\|^{2} }}$, where *γ* is the kernel parameter. The *γ* parameter was tuned by trial and error. The Gaussian radial basis function satisfied by the SVR kernel and is described as the relation: $k({\mathbf{x}},{\mathbf{x}}_{\text{i}} ) = \varphi^{T} ({\mathbf{x}})\varphi ({\mathbf{x}}_{\text{i}} )$. The optimal value of the *ε* (i.e., insensitive loss function) parameter was determined after a series of experiments and was assumed to be *ε* = 0.001.

The test was performed under the simulation mode (i.e., estimated output is used to form the regressors). Both the sets of parameters and excitation signals of input were different from those in the training and validation mode. The procedure for testing the data collection was the same as for the training and validation mode. The number of SVR inputs (i.e., the feature vector) was the same as in the training and validation mode. The comparison of the PID algorithm’s output and SVR’s output was for the same sets of parameters. In both controllers, the anti-windup was turned on.

**Fig. 14:**
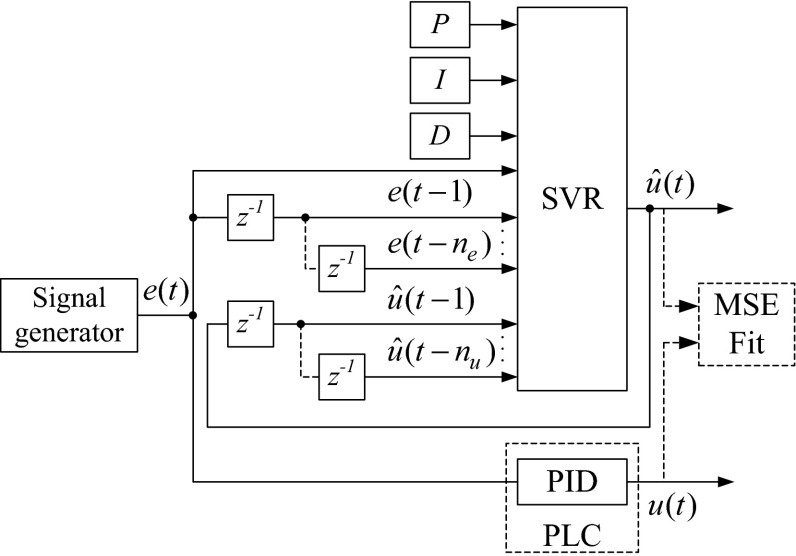
Diagram of the test procedure all structures

The diagram of the procedure for the test of the SVR and comparison of the accuracy Fit and MSE for any setting *P*, *I*, and *D* are shown in Fig. 14.

The signal *e* was varied over the range of −1 to 1 V. The output of the SVR and PID algorithm implemented in the PLC was compared at similar time points, and the MSE and Fit error were calculated. The test procedure was conducted in accordance with Fig. 14 and ten times repeated for different sets. The final results of the SVR, transfer function of the PID and NN test were averaged from several tests.

**Fig. 15:**
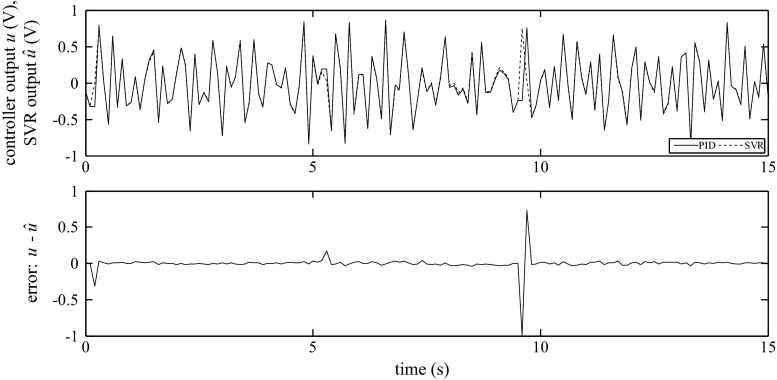
Output of the PID algorithm (PLC GE VersaMax ‘PID IND’ function block) as well as the corresponding simulated outputs of the SVR for *K*_P_ = 0.41, *K*_I_ = 0.21, *K*_D_ = 0.48

**Fig. 16:**
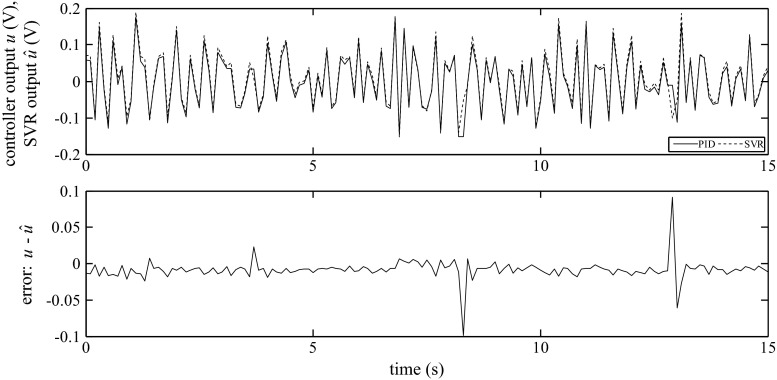
Output of the PID algorithm (PLC GE VersaMax ‘PID ISA’ function block) as well as the corresponding simulated outputs of the SVR for *K*_C_ = 0.66, *T*_I_ = 5.8, *T*_D_ = 0.1

**Fig. 17:**
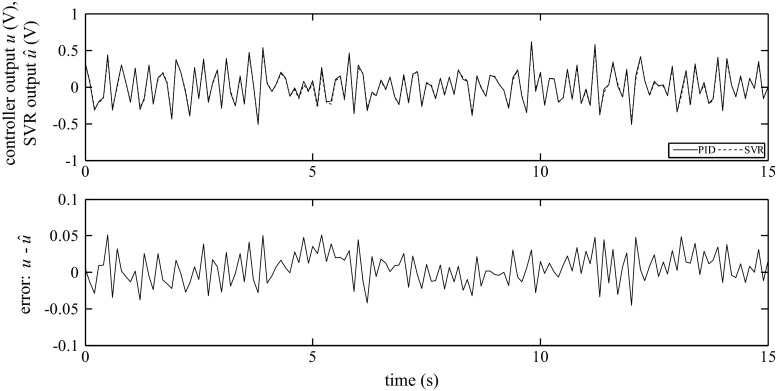
Output of the PID algorithm (PLC Siemens ‘FB41’ function block) as well as the corresponding simulated outputs of the SVR for *K*_C_ = 0.84, *T*_I_ = 6.5, *T*_D_ = 0.32

The examples of the SVR responses and the comparison with the response of the real output of the PID algorithm for Siemens and GE for all examined structures are shown in Figs. 15, 16, and 17.

As shown in Figs. 15, 16, and 17, the outputs of the SVR coincide with the response of the PID algorithms implemented in the PLCs. It should be noted that this option is a considerably more difficult task than the one-step-ahead prediction. In this case, there is a risk of cumulative error because the inputs and estimated output are used to form the regressors.

The final results of the training and testing of the SVR combined with the results for the transfer function of PID algorithms implemented in the PLC and NNs for both Siemens and GE are shown in Tables 1, 2, and 3.

**Table 1:** Results for the SVR, Tf model and NNs, parallel form GE

	MSE (−)		Fit (%)	
	Training set	Test set	Training set	Test set
SVR: C = 100 *γ* = 2.79 × 10^−2^	4.6 × 10^−4^	9.1 × 10^−4^	90.93	86.96
Tf of PID	–	1.5 × 10^−3^	–	83.52
NN^a^ 14-5-1	1.2 × 10^−3^	2.9 × 10^−3^	84.89	80.14

^a^Where NN 14-5-1 means: 14 is the number of inputs; five is the number of hidden neurons; one is the number of outputs

**Table 2:** Results for the SVR, Tf model and NNs, ISA GE

	MSE (−)		Fit (%)	
	Training set	Test set	Training set	Test set
SVR: C = 150 *γ* = 3.14 × 10^−2^	2.4 × 10^−4^	4.0 × 10^−4^	91.26	88.62
Tf of PID	–	6.5 × 10^−4^	–	88.18
NN 14-5-1	9.0 × 10^−5^	1.9 × 10^−3^	94.51	81.07

**Table 3:** Results for the SVR, Tf model and NNs, ISA Siemens

	MSE (−)		Fit (%)	
	Training set	Test set	Training set	Test set
SVR: C = 120 *γ* = 3.04 × 10^−2^	1.5 × 10^−4^	2.8 × 10^−4^	92.00	89.67
Tf of PID	–	4.6 × 10^−4^	–	87.67
NN 13-7-1	2.8 × 10^−4^	2.4 × 10^−3^	90.18	83.35

As shown in Tables 1, 2, and 3, the results of both the MSE and Fit methods for the training and testing of the SVR are better than the Tf and NNs for the PID algorithms implemented in both PLCs. The results of the SVR for the ISA (Fit—88.62, 89.67 %) are better compared to that of the parallel form (86.96 %). It can be assumed that the parallel form may be slightly modified, as confirmed by the worse result for the transfer function of the PID algorithm (83.52 %). The results for the ISA for both Siemens (89.67 %) and GE (88.62 %) are similar, most likely due to the similar implementation of the algorithms in the PLC. Generally, good results for the Tf (83.52, 88.18, 87.67 %) indicate for small modifications introduced by manufacturers. The worst results obtained for the NN (80.14, 81.07, 83.35 %) are clear because learning algorithms can stop at the local minimum. For the SVR, the optimization process is convex and has a single optimum value. Furthermore, the minor difference between the results of the training and testing of the SVR (90.93–86.96 % for parallel form GE, 91.26–88.62 % for ISA GE, 92.00–89.67 % for ISA Siemens) demonstrates good generalization properties.

## Conclusions and future work

The goal of this study was to demonstrate a method that improves the accuracy of a PID algorithm simulation by considering the real dynamics of control algorithms. We have shown the practical advantages of the SVR and that it is better to simulate the PID algorithm using SVR than the ready transfer function of PID algorithms provided by the manufacturer. The manufacturer often provides only a general transfer function of PID algorithms and does not inform on additional functions or modifications. Furthermore, we show that the SVR maps the function of PID algorithms with the modifications introduced by the PLC manufacturer with high accuracy. In this report, only the most frequently implemented PID structures were studied. However, this approach can be extended to any structure due to the application of black box modeling, which does not require knowledge of the structure or the modifications provided by the manufacturer. With this approach, the SVR simulation results can be used to tune the PID algorithms in the PLC. In additional studies, we intend to focus on emulating additional functions, e.g., anti-windup, control zone, deadband, slew time, and weakening proportional action as well as their combinations. Furthermore, we intend to use ICA in connection with SVR, which will enable a quick and accurate tuning of different PID algorithms used in real PLCs from any manufacturer, and to compare the results with auto-tuning implemented in the PLC.
